# Modulating commensal bacteria to intervene in cancer immunotherapy

**DOI:** 10.1002/ctm2.1704

**Published:** 2024-05-22

**Authors:** Dingjiacheng Jia, Liangjing Wang

**Affiliations:** ^1^ Department of Gastroenterology Second Affiliated Hospital of Zhejiang University School of Medicine Hangzhou China; ^2^ Institution of Gastroenterology Zhejiang University Hangzhou China; ^3^ Cancer Center Zhejiang University Hangzhou China

Various immune checkpoint inhibitors such as anti‐programmed cell death protein 1 antibody (αPD‐1) have been used in the clinic, but the associated low response rate and high incidence of side effects have hindered their further use. Gut microbiota has been widely identified as a potential adjunct to sensitizing immunotherapy.^1^ The microbial composition of faeces varies in patients with different sensitivities to immunotherapy, and the efficacy of immunotherapy can be influenced by faecal microbiota transplantation or supplementation with specific strains. We recently reported in *Cell* that a gut microbiota derivative, indole‐3‐propionic acid (IPA), increases the responsiveness of cancer immunotherapy by modulating T‐cell stemness.^2^

We first identified the ‘Responder’ and the ‘Poor‐responder’ mice in mice receiving immunotherapy similar to clinical practice. The gut microbiota of these mice was found to be different by microbiome sequencing, leading us to speculate that gut microbiota might be involved in cancer immunotherapy response. We also found that mice transplanted with the ‘Poor‐responder’ faeces were less responsive to immunotherapy and screened a strain of *Lactobacillus johnsonii* (*L. johnsonii*) (Figure 1A). Gavage administration of *L. johnsonii* to mice increased CD8^+^ T cell infiltration to sensitize the efficacy of αPD‐1 therapy.

**FIGURE 1:**
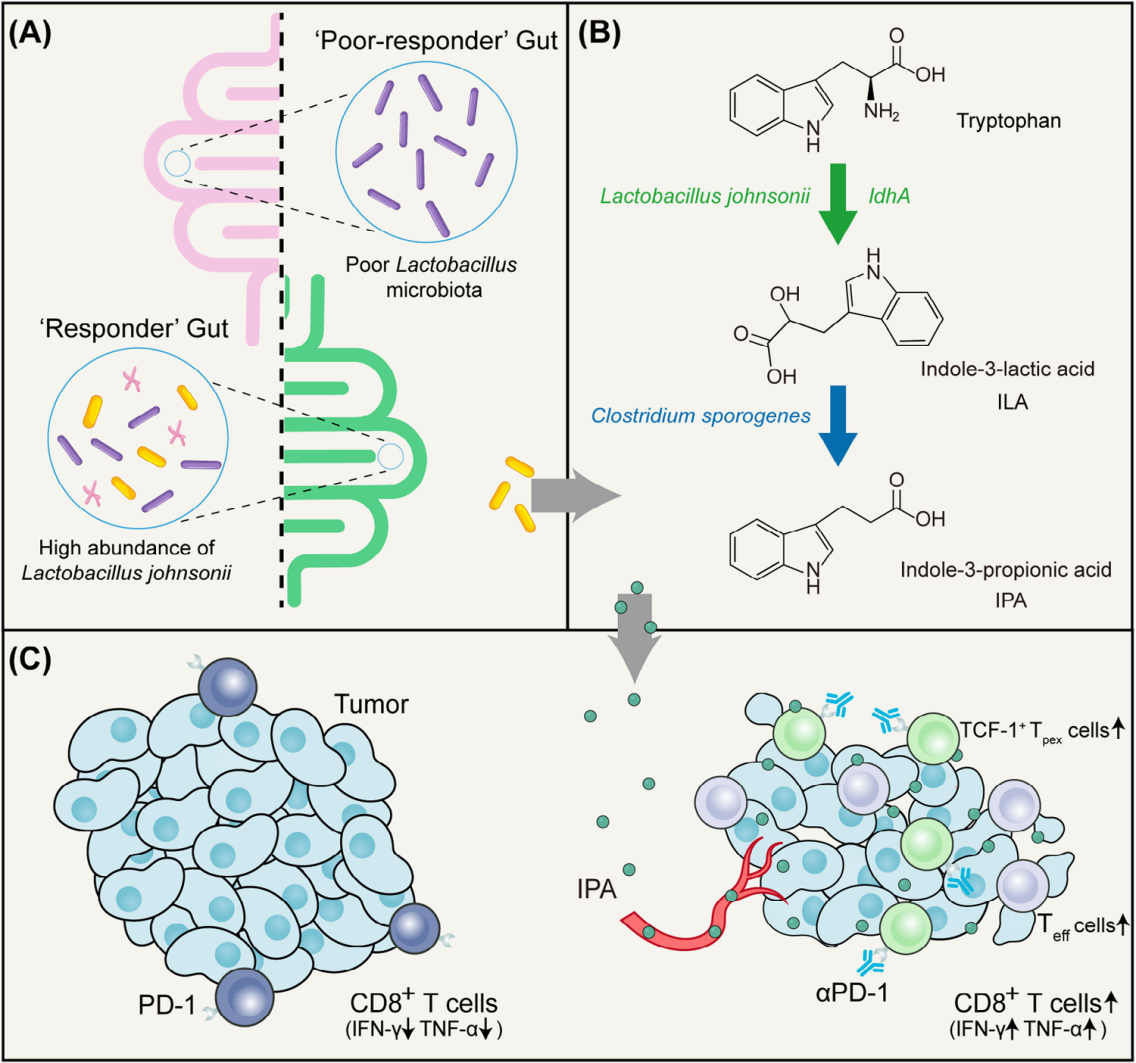
**Microbial metabolite enhances immunotherapy efficacy by modulating T‐cell stemness in pan‐cancer**. (A) Patients with a poor response to immunotherapy have a lower abundance of *Lactobacillus* in the gut, whereas the gut of responding patients contains higher levels of *Lactobacillus johnsonii*. (B) *Lactobacillus johnsonii* converts tryptophan to indole‐3‐lactic acid (ILA) via the enzyme *ldhA*, and *Clostridium sporogenes* further converts ILA acid to indole‐3‐propionic acid (IPA). (C) Indole‐3‐propionic acid (IPA) modulates the stemness programme of CD8^+^ T cells and promotes the generation of progenitor‐exhausted CD8^+^ T cells (T_pex_) and effector CD8^+^ T cells (T_eff_), thereby improving immune checkpoint blockade responsiveness.

A growing number of specific gut strains have been shown to play a role in immunotherapy, but they act with different components, some through their own bacterial components and others through secreted metabolites.^3^, ^4^, ^5^ There is a lack of specific means and standardized procedures to reveal the exact components of bacterial action. We used different treatments to investigate the active components of *L. johnsonii*. Compared with heat‐inactivated and ultrasonically fragmented bacteria, the culture supernatant of *L. johnsonii* had an effect similar to that of live bacteria, suggesting that *L. johnsonii* colonized in the gut may secrete relevant metabolites into the blood. In combination with non‐targeted plasma metabolic mass spectrometry and the tryptophan‐deficient diet, we identified a key indole derivative: IPA. Gavage of IPA also enhanced the efficacy of αPD‐1 by increasing CD8^+^ T cell infiltration and effector secretion.

As an essential amino acid, tryptophan is metabolized by three main pathways: the 5‐hydroxytryptamine pathway, the kynurenine pathway and the indole pathway. Of these, the indole pathway is relatively unique in that it is mainly carried out by intestinal microorganisms.^6^ The gut microbiota genome contains a large number of unknown metabolic enzyme gene clusters that are capable of performing a wide range of metabolic reactions that cannot be performed by the host alone. A number of specific bacteria, such as *Lactobacillus*, *Bifidobacterium* and *Clostridium*, have been identified as containing key enzymes that stepwise breakdown tryptophan into a variety of indole derivatives.^7^ Recent studies have also focused on the role of indole derivatives in regulating the physiological functions of organs and in the development of disease. Among these, IPA has been shown to promote nerve regeneration and repair,^8^ prevent ejection fraction and heart failure,^9^ and prolong survival in rats with traumatic colon injury,^10^ but its role in cancer immunotherapy has not yet been clarified.

We further used *Rag1* ^−/−^ mice, anti‐CD8 neutralizing antibody, and adoptive cell transfer therapy to validate the sensitizing immunotherapeutic effects of *L. johnsonii* and its derived IPA dependent on CD8^+^ T cells, and sorted CD8^+^ T cells infiltrated in tumours of mice for single‐cell RNA sequencing (scRNA‐seq), single‐cell TCR sequencing (scTCR‐seq) and single‐cell assay for targeting accessible‐chromatin with high‐throughout sequencing (scATAC‐seq). Gavage IPA significantly increased the proportion of ‘stem cell‐like’ progenitor‐exhausted CD8^+^ T (T_pex_) cells and effector CD8^+^ T cells. These two subsets of CD8^+^ T cells shared TCR profiles and underwent clonal expansion. Combined with single‐cell transcriptomic pathway enrichment and scATAC‐seq, we found that IPA regulates chromatin opening in T_pex_ cells through histone acetylation modifications. Western blot, ChIP, CUT & RUN and CUT & Tag sequencing further revealed that IPA increases H3K27 acetylation in the *Tcf7* super‐enhancer region.

Recent studies have shown that T_pex_ cells with high expression of the transcription factor T‐cell factor 1 (TCF‐1, encoded by *TCF7*) and immunocyte stemness properties are a key subset responding to immunotherapy with anti‐PD‐1 antibodies.^11^ Further activation of T_pex_ cells to increase immune cell stemness could be an important strategy to improve the efficacy of immunotherapy. *Tcf7* knockout mice are associated with an imbalance in gut microbiota,^12^ but the ability of specific strains and their metabolites to modulate the immunocyte stemness has not been reported. Here, we found that bacterial‐derived IPA has the potential to regulate T‐cell stemness and *Tcf7* ^−/−^ mice verified that IPA requires T_pex_ cells for sensitizing immunotherapeutic effects.

We then explored how *L. johnsonii* produces IPA. Interestingly, IPA was not detected in *L. johnsonii* culture supernatants in vitro, but instead, the upstream metabolite of IPA, indole‐3‐lactic acid (ILA) was detected. Based on the antibiotic cocktail model and intraperitoneal injection model, we hypothesized that ILA produced by *L. johnsonii* is further metabolized to IPA by other gut microbiota in vivo and verified this in germ‐free mice model. Colonization of germ‐free mice with *L. johnsonii* alone produces only ILA, whereas mixed administration of *L. johnsonii* and *Clostridium sporogenes*, produces large amounts of IPA, sensitizing the efficacy of immunotherapy.

The intestinal microbiota can be regarded as a small society, the members of which are combined in a certain proportion, mutually restrained, dependent on each other, and form a kind of ecological equilibrium both qualitatively and quantitatively. Many bacterial metabolites are not produced by a single bacterium, and there is complex cooperation and competition between gut microbes at the genus level, at the species level, and even between strains. Recent publications have focused on the synthetic processing of metabolites by means of cooperation between bacteria. *C. scindens* cooperates with its related strain to convert primary bile acids to secondary bile acids.^13^ *Bacteroides* cooperate with *Lactobacillus plantarum* to metabolize host tyrosine.^14^ We provide an additional paradigm that *L. johnsonii* and *Clostridium sporogenes* could work together to metabolize tryptophan to biosynthesize IPA (Figure 1B).

We also predicted that *ldhA* was the key enzyme for ILA production by *L. johnsonii* and transformed a ldhA expressional vector into *E. coli*. *E. coli* overexpressing *ldhA* increased IPA production and sensitized immunotherapeutic effects. Finally, we verified the regulatory effects of IPA on T_pex_ cells and immunotherapy in a variety of mouse tumour models (both breast cancer and melanoma transplantable tumour model, mammary fat pad orthotopic implantation model, MMTV‐PyMT spontaneous breast cancer model and cecum orthotopic implantation model) and in organoids derived from colorectal cancer patients (Figure 1C).

In summary, this work reveals a tryptophan derivative cooperatively produced by different bacteria, elucidates the mechanism by which IPA regulates key transcription factors of immune stemness through histone acetylation modifications, and validates the sensitizing immunotherapeutic effects of IPA at the pan‐cancer level. This bacterial‐derived metabolite‐host immunomodulatory pathway provides a potential idea for sensitizing cancer immunotherapy. For future clinical applications of gut microbiota, it is necessary to consider the ability of bacteria to colonize efficiently and produce metabolites in a sustained manner. Particularly for non‐native bacteria, it may be necessary to construct engineered bacteria by homologous recombination to achieve efficient metabolite synthesis. Recent publications have also highlighted the potential of gut microbes to mitigate adverse effects associated with immune checkpoint inhibitors,^15^, ^16^ which is likely to be a future research trend. Overall, although larger, multi‐centre clinical trials are needed to clarify the role of the gut microbiota in cancer immunotherapy, it certainly has a promising future.

## AUTHOR CONTRIBUTIONS

Writing—Original Draft, Dingjiacheng Jia; Writing—Review & Editing, Liangjing Wang.

## CONFLICT OF INTEREST STATEMENT

The authors declare no conflict of interest.

## FUNDING INFORMATION

The National Foundation of Natural Science of China (82273269 to L.‐J.W.).

## ETHICS STATEMENT

Not applicable.
